# Modelling Human Hair Follicles—Lessons from Animal Models and Beyond

**DOI:** 10.3390/biology13050312

**Published:** 2024-04-30

**Authors:** Chew Teng Tan, Chin Yan Lim, Kenneth Lay

**Affiliations:** 1 A*STAR Skin Research Labs (A*SRL), Agency for Science, Technology and Research (A*STAR), 8A Biomedical Grove, #06-06 Immunos, Singapore 138648, Singapore; 2 Department of Biochemistry, Yong Loo Lin School of Medicine, National University of Singapore, Singapore 117596, Singapore

**Keywords:** human hair models, hair follicle development, signaling pathways

## Abstract

**Simple Summary:**

Hair follicles are mini-organs in the skin that have regenerative cycles of growth, regression, and rest. The disruption of each phase in the hair cycle may cause hair-related issues such as hair loss. These defective events that occur in the hair cycle are well-studied in mice, but they are pending validation in humans due to limitations of current in vitro human hair models, whose improvement will add significant value to human hair research and hair loss treatment. However, it has been challenging to recreate the structural organization of highly diverse cell types that make up the hair follicle and its surrounding tissue in vitro. This review discusses the current knowledge of hair follicle formation and efforts to bioengineer hair follicles or hair-bearing skin in vitro.

**Abstract:**

The hair follicle is a specialized appendage of the skin that is critical for multiple functions, including thermoregulation, immune surveillance, and sebum production. Mammals are born with a fixed number of hair follicles that develop embryonically. Postnatally, these hair follicles undergo regenerative cycles of regression and growth that recapitulate many of the embryonic signaling pathways. Furthermore, hair cycles have a direct impact on skin regeneration in homeostasis, cutaneous wound healing, and disease conditions such as alopecia. Here, we review the current knowledge of hair follicle formation during embryonic development and the post-natal hair cycle, with an emphasis on the molecular signaling pathways underlying these processes. We then discuss efforts to capitalize on the field’s understanding of in vivo mechanisms to bioengineer hair follicles or hair-bearing skin in vitro and how such models may be further improved to develop strategies for hair regeneration.

## 1. Background

**Figure 1:**
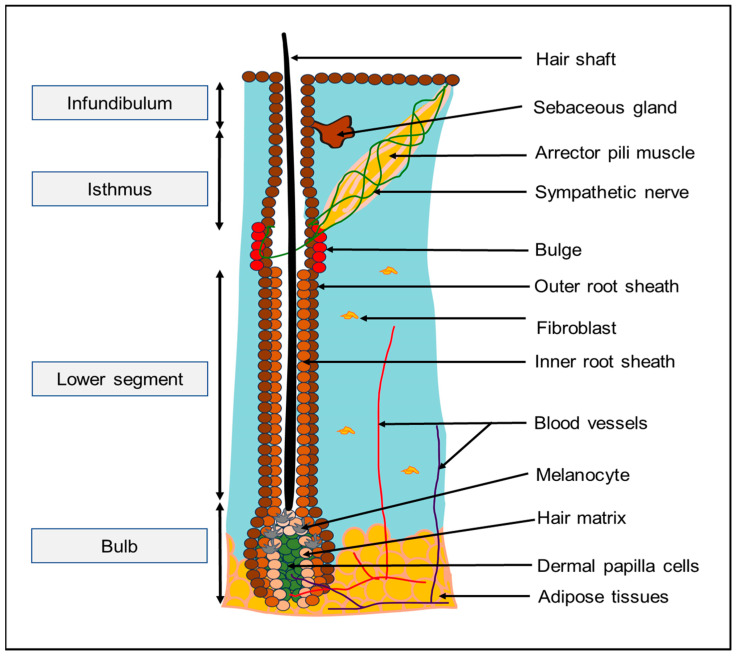
The pilosebaceous unit consists of the arrector pili muscle, sebaceous gland, and hair follicle.

The hair follicle is part of the pilosebaceous unit which also consists of the sebaceous gland and arrector pili muscle. It is made up of cells of both epithelial and mesenchymal origins and comprises the infundibulum (uppermost region that connects it to the interfollicular epidermis), bulge (where hair follicle stem cells [HFSCs] reside), isthmus (which connects the sebaceous gland and bulge), lower follicle, and bulb (where progenitor cells divide and differentiate into its specialized lineages) (Figure 1) [1,2]. Together with other parts of the pilosebaceous unit, the hair follicle serves as a sensory organ that protects us from the external environment through thermoregulation, immune surveillance, and sebum production [1,2,3]. It also plays important functions in skin regeneration by supporting pigmentation [4,5,6], as well as wound healing processes, including epidermal re-epithelialization [7,8,9,10] and dermal remodeling [11,12].

**Figure 2:**
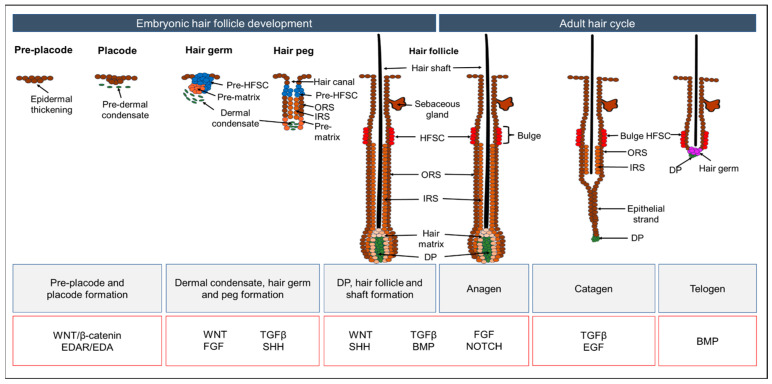
Embryonic hair follicle development and adult hair cycle, and the main signaling pathways involved. BMP, bone morphogenetic protein; DP, dermal papilla; EDA, ectodysplasin; EDAR, ectodysplasin receptor; EGF, epidermal growth factor; FGF, fibroblast growth factor; HFSCs, hair follicle stem cells; IRS, inner root sheath; ORS, outer root sheath; SHH, sonic hedgehog; TGFβ, transforming growth factor beta.

Hair follicles are maintained by undergoing cyclical phases of growth, regression and rest known as the hair cycle (Figure 2). The growth phase, also called “anagen”, is primarily driven by hair follicle stem cells and their progenitors that proliferate and differentiate in response to their major signaling centre, the dermal papilla (DP), to give rise to elongating hair follicles and hair shafts. After a period of active growth, hair follicles undergo “catagen”, a regressive phase in which most of the lower follicles below the bulge die by apoptosis. Thereafter, hair follicles enter a resting dormant phase known as “telogen”, before re-initiating a new round of anagen [1,13,14]. A fourth phase that is also actively regulated but occurs independently of the hair cycle is “exogen”, in which the hair fiber is shed from the follicle [15].

Alopecia, a condition in which excessive hair loss occurs, stems from dysfunctional hair follicles. Two common forms of alopecia are androgenetic alopecia (AGA) and alopecia areata (AA). AGA is caused by excessive activation of androgen receptors (AR) in the DP [1]. This in turn causes progressively shorter anagen, prolonged telogen, and eventually hair follicle miniaturization. Increased AR activity is a consequence of increased AR expression, mutations in the AR gene, and/or increased DP expression of Type II 5-alpha-reductase, which converts testosterone to dihydrotestosterone (DHT) that then stimulates AR with higher affinity than testosterone [16,17,18,19,20]. On the other hand, AA is an autoimmune disease whose key feature is immune cells attacking the anagen hair follicle bulb, inducing its premature entry into catagen and causing bald patches on the scalp and other body sites [21,22]. Other causes of alopecia include aging, physical trauma, infections, stress, medications, and chemotherapy [23]. While not life-threatening, alopecia can be emotionally and psychologically stressful. Being able to restore hair growth will vastly improve health outcomes. This necessitates a deep understanding of the biological mechanisms underlying hair follicle development, homeostasis, neogenesis, and disease pathogenesis that can then be deployed both in vivo and in vitro.

Mice have been classically employed as model systems to understand hair follicle biology. Murine hair follicles, like their human counterparts, also undergo cycles of growth, regression, and rest. In addition to their high genetic similarity to humans, mice can be easily manipulated by gene editing tools, allowing the study of genes through loss- and gain-of-function with precise spatial and temporal control. Further, with the skin being the largest and most accessible organ, hair follicle activity can be conveniently tracked by simply observing hair coat recovery and wound healing capabilities [2]. However, apparent differences in hair cycle durations and hormonal responses exist between mouse and human hair follicles (discussed further in Section 2.3 below) to preclude the direct translation of findings in mice to therapeutic strategies for human hair loss conditions [24].

Therefore, there is an urgent need for sophisticated in vitro models of human hair follicles to deepen our understanding of their induction, development, and regeneration, as well as to elucidate disease mechanisms. Here, we review the current knowledge of hair follicle formation during embryonic development and post-natal hair cycles, with an emphasis on the underlying molecular signaling pathways. We then discuss efforts to capitalize on the field’s understanding of in vivo mechanisms to bioengineer hair follicles or hair-bearing skin in vitro. Finally, we will share our insights on how we can translate knowledge gained from mouse models into the establishment of human models for biological and therapeutic discoveries.

## 2. Hair Follicle Development and Regeneration 

### 2.1. Signaling Pathways

Between mouse embryonic day 9.5 (E9.5) and E12.5, the skin epidermis exists as a single layer of ectoderm-derived epithelial cells and begins to establish signaling crosstalk with its underlying mesenchymal cells that originate from either neural crest (in the craniofacial region) or mesoderm (in the rest of the body) (Figure 2). At ~E13.5, some of these epithelial cells respond to mesenchymal WNT signaling and become specified to form hair placodes in a spatially organized manner. WNT signaling is critical to this process, as mice that express high levels of WNT inhibitor Dickkopf 1 (DKK-1), or lose downstream WNT signaling effectors such as β-catenin and LEF1, display an early and complete block in hair follicle development [25,26,27,28]. In addition to WNT, several other signaling pathways are also required for hair follicle induction. WNT signaling induces the expression of EDAR in hair placodes. In turn, EDA/EDAR signaling leads to downstream NF-κB signaling, which triggers transcriptional regulation for hair placode development [29,30]. FGF20 is the only “first epithelial signal” identified thus far that specifies mesenchymal fibroblasts to migrate, aggregate, and form dermal condensates (DC) beneath individual hair placodes [31]. While WNT, EDA/EDAR, and FGF20 signals are active, BMP signaling must be inhibited to allow for hair follicle specification. Dermal SHH signaling maintains DC expression of the BMP inhibitor, Noggin, which promotes placode formation [32]. At the same time, DC-derived BMP4 and placode-derived BMP2 act to inhibit surrounding interfollicular epithelial cells from taking on a hair follicle fate, thereby maintaining proper spacing between developing hair follicles [33].

Continuous epithelial–mesenchymal crosstalk remains essential for hair placodes to subsequently invaginate into the dermis, downgrow, and develop into hair germs, hair pegs, and eventually hair follicles. Here, SHH and TGFβ signaling are crucial, as loss of either signaling pathway arrests hair follicle invagination and downgrowth [32,34,35,36,37]. SHH signaling, driven by high WNT activity in basal placode cells, is also essential to induce symmetric division of the overlying suprabasal cells. These suprabasal placode cells, marked by high SOX9 expression, are set aside as HFSCs that will drive future hair cycles during adulthood [38]. As hair follicles downgrow, the DC becomes encased by epithelial cells and matures into a DP. In contrast to its earlier role in inhibiting hair follicle fate, active BMP signaling in the DP is now pivotal for it to instruct surrounding epithelial cells to differentiate into various lineages of the mature hair follicle, including hair shaft and inner root sheath layers [39].

Many of these developmental signaling pathways are subsequently re-employed by post-natal and adult hair follicles during homeostasis. BMP signaling originating from the dermis, subcutaneous adipose tissue, and inner bulge layer of differentiated cells, is key to maintaining hair follicle stem cell quiescence during telogen [14,40]. As in embryonic development, inhibition of BMP signaling through DP-derived noggin, along with WNT, TGFβ, and FGF signaling, are key events that initiate anagen and hair follicle downgrowth by sequentially triggering hair germ and bulge HFSCs to proliferate and give rise to the matrix and outer root sheath (ORS) respectively [13,14,40,41,42]. SHH signaling originating from the matrix reinforces the proliferative state of both the matrix and ORS and drives hair follicle elongation [43]. BMP and WNT signaling in the DP then orchestrate the differentiation of matrix transit amplifying cells (TACs) into various layers of the mature hair follicle [44,45,46]. 

### 2.2. Complex Regulation of Hair Cycling beyond Epithelial-Mesenchymal Cross-Talk

Hair cycling in mice is increasingly known to be heavily influenced by various other constituents of the skin microenvironment. A distinct subset of TREM2+ macrophages in the dermis has been identified as producing Oncostatin M (OSM), which binds its receptor and induces JAK-STAT5 signaling in HFSCs to maintain their quiescence [47]. On the other hand, regulatory T cells (Tregs) preferentially reside close to the telogen bulge where they induce Notch signaling in HFSCs and promote their activation [48]. Likewise, intradermal adipocyte precursor cells produce PDGFA that acts via the DP to induce HFSC activation [49].

Bulge HFSCs themselves also influence their niche to regulate their activity. They share their niche and tightly coordinate their activity with melanocyte stem cells, which differentiate into melanocytes to result in hair pigmentation during hair cycling [6,50]. During telogen, HFSCs produce ANGPTL7, promoting their physical association with lymphatic vessels which contribute to their quiescence. During early anagen, HFSCs produce ANGPTL4 instead. This causes lymphatic vessels to become dissociated from the bulge, allowing for HFSC activation [51]. In addition to lymphatic vessels, blood vessels that span under hair follicles also act to reinforce HFSC quiescence and telogen through the production of BMP4. During early anagen, bulge HFSC-derived hair germ cells express the transcription factor RUNX1, which acts to reduce vasculature density, thereby creating a more permissive environment for HFSC activation [52].

To perform their function as a sensory organ, hair follicles are richly innervated. Nerves, in turn, have also been found to be essential constituents of the HFSC niche. Sensory nerves are in contact with the upper part of the bulge and are a source of SHH. SHH signals to HFSCs specifically in this region of the bulge and enables them to take on an interfollicular epidermal cell fate and contribute to re-epithelialization during wound healing [53]. On the other hand, sympathetic nerves loop around the arrector pili muscle (APM) that is connected to the HF bulge, and form synapse-like connections with HFSCs, allowing hairs to “stand on end” upon environmental stimuli, nerve stimulation, and APM contraction. Sympathetic nerve-derived norepinephrine serves to modulate HFSC activity by keeping them poised for activation. Upon stimulation, sympathetic nerves trigger HFSCs and HFs to enter anagen. In fact, HFs already play a role in shaping their future stem cell niche from very early on when developing HFs express SHH to regulate APM development and sympathetic innervation to future HFSCs [54].

### 2.3. Differences in Mouse and Human Hair Biology 

Much of our afore-described knowledge in hair follicle development and homeostasis originated from studies in mice. How much of this is recapitulated in humans remains to be validated, as human and murine hair follicles do show apparent differences. In humans, lanugo hair is the first hair type that is made during development. This becomes replaced by vellus and terminal hairs towards the end of gestation and postnatally, with vellus hairs at certain body sites becoming terminal hairs in response to androgens during puberty. In mice, there are at least eight different hair types, including vibrissae (whiskers), tail hair, and dorsal guard, awl/auchene, and zigzag hairs, all of which originate from embryonic development and persist into adulthood [2]. 

The hair cycle in humans also differs from that in mice. Human scalp hair follicles largely exist in anagen, which lasts several years and is comparatively longer than telogen (typically 2–3 months, becoming progressively longer with age). In contrast, mouse dorsal hair follicles largely reside in telogen, which lasts for months, rather than in anagen, which is only 2–3 weeks long [55,56].

Gene signatures that have been characterized for various mouse hair follicle regions, in particular, bulge HFSCs (CD34+, Keratin 15 [K15]+) [57,58,59], might not be fully recapitulated in humans (putative stem cells are CD34− but K15+ and CD200+) [60,61,62]. While the signaling pathways governing mouse hair follicle development and cycling are well-elucidated, many of these still require investigation and validation in humans. Importantly, mouse hair follicles respond to hormones differently from human hair follicles. Estrogen and prolactin inhibit mouse anagen but prolong human anagen [63,64]. On the other hand, androgens do not elicit hair loss in mice like they do in humans [65,66]. These distinctions have hampered efforts to model human hair loss conditions in mice, such as AGA which is caused by excessive AR activation in human DP, and to translate findings from mouse models into therapeutic strategies.

## 3. Current In Vitro Human Hair Models

**Figure 3:**
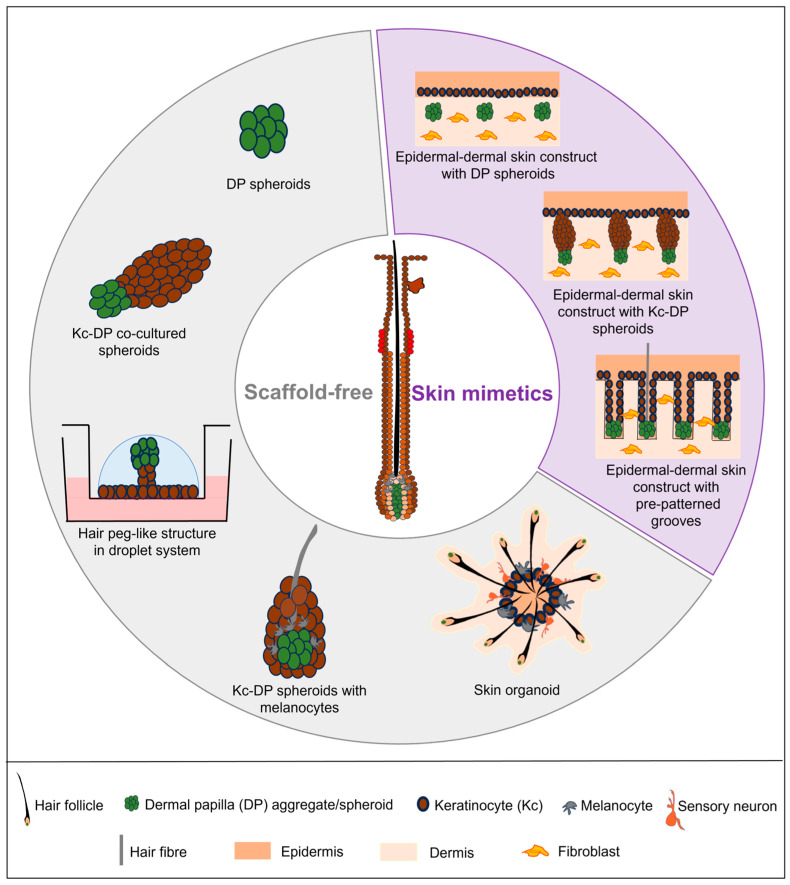
Overview of current in-vitro hair models, cultured either in scaffold-free conditions (depicted in grey sector) or on skin mimetics (depicted in purple sector). Scaffold-free models include dermal papilla (DP) spheroids [67,68], keratinocyte (Kc)-DP co-cultured spheroids [69], hair peg-like structure in droplet system [70], Kc-DP spheroids with melanocytes [71], and skin organoid [72]. Skin mimetic models are based on epidermal-dermal skin constructs with DP spheroids [73], Kc-DP spheroids [74] or pre-patterned grooves [75].

There is keen interest in creating models of human hair follicles to facilitate studies of human hair morphogenesis and regeneration in normal and aging homeostatic or disease conditions. Efforts to generate human hair follicles or hair-bearing skin models involve understanding and replicating, in full or partially, the complex cellular interactions and inductive signaling in the hair follicle niche in vitro. Existing models can be broadly classified into two categories: those that are cultured in free-floating, scaffold-free conditions, and those that are cultured on skin-mimetics (Figure 3). 

### 3.1. Scaffold-Free Models

#### 3.1.1. DP Spheroids

Reciprocal signaling between the DP and hair follicle progenitor cells, including TGFβ2, Noggin, FGF7, FGF10, and BMP signals, are required for hair follicle generation [13,39,41]. Maintaining the ability of DP cells to induce hair follicle formation and differentiation is, therefore, a key goal of any effort to generate in vitro hair follicle models. Human DP cells, typically isolated from fetal or adult scalp follicles, rapidly lose inductive capacity in culture. DP inductivity was shown to be better preserved when cells are cultured as 3D spheroids rather than 2D monolayers [67]. Dissociated DP cells made to aggregate in hanging drops reactivated the expression of key signature genes such as *BMP2*, *HEY1*, *FGF7*, and *SFRP2* [67,68]. DP spheroids formed by aggregating isolated adult DP cells, but not DP cells maintained in 2D cultures, were found to induce hair follicle formation when implanted onto the dorsal skin of nude mice [68,76]. Extensive research has since gone into improving the hair-inducing properties of cultured DP spheroids by culturing them in keratinocyte-conditioned media [76] or supplementing culture media with extracellular matrices (ECM). These include natural additives such as Matrigel [77] and synthetic ECM such as silk-gelatin [78], self-assembling peptide scaffold [79], and fibrin-microgel derived from blood plasma [80]. These strategies could enhance BMP and WNT/β-catenin signaling [78], improve cellular functions such as proliferation and migration [77,78], induce a secretome profile that resembles that of anagen DP [76], and support the cellular organization and viability of DP cells by further promoting their production of other ECMs such as collagen IV and versican [79,80]. 

#### 3.1.2. Co-Culture of DP Spheroids with Keratinocytes and Melanocytes 

Capitalizing on these observations, 3D co-cultures of human keratinocytes and DP cells were developed in attempts to induce hair follicle formation in vitro. In the simplest system, dissociated skin epithelial keratinocytes and DP cells were co-cultured in low-attachment wells or 3D droplets to facilitate direct cell interaction and cross-signaling [69,70]. Cells in these mixed aggregates spontaneously re-organized into distinct DP and keratinocyte compartments in 3D structures that resembled hair germs or pegs. However, while the polarization of spheroids occurred within days, no further hair follicle development was observed with longer times in culture. Further development of immature hair pegs into cellularly complex hair follicles may require additional signaling factors that are absent from typical keratinocyte-DP co-cultures. To test this, researchers have added in growth factors known to contribute to in vivo hair follicle development, including SHH, TGFβ2, FGF2, FGF7, FGF10, and WNT7A [70]. However, these attempts still did not result in fully mature hair follicle formation in vitro.

Of note, keratinocytes derived from neonatal or adult interfollicular epidermis were used in these keratinocyte-DP co-culture systems [69,70]. Greater success was achieved when cells derived from hair follicles were used instead. Three-dimensional co-cultures of outer root sheath keratinocytes, melanocytes, and DP cells, in proportions similar to that in early anagen follicles, were shown to result in hair fiber formation [71]. The findings that follicle-derived keratinocytes could support the formation of mature hair follicles suggest that the in vitro culture conditions lack essential factors that can fully drive the re-specification of epidermal keratinocytes into the follicular lineage [10]. The inclusion of more cell types in the co-cultures, such as melanocytes, which have been shown to participate in cross-talk with epithelial cells in mice, may provide the necessary additional signaling [6,50]. However, obtaining a single media formulation that is compatible with the survival and growth of different cell types is challenging and a major deterrent for co-cultures of multiple cell types. Taken together, these studies demonstrate a requirement for the programmability of keratinocytes in response to the complex signaling within the microenvironment for hair follicles to form in vitro. 

#### 3.1.3. Skin Organoid

Lee and colleagues overcame these limitations by using pluripotent stem cells that can differentiate into cells of all three embryonic lineages, and successfully generated hair-bearing skin organoids [72] with a protocol that has been successfully adopted by others [81,82,83,84,85]. Through stepwise addition of growth factors, they coaxed human embryonic stem cells (ESCs) and induced pluripotent stem cells (iPSCs) to form skin organoids, first by inducing development of epidermis (BMP4, TGFβ-inhibitor), followed by cranial neural crest cells that gave rise to dermis (FGF2, BMP-inhibitor), thereby creating spatial and temporal cues that closely mimic embryonic skin development. Over time, hair placodes formed and developed into lanugo hair follicles within these skin organoids, which notably comprised various other skin cell types as well, including melanocytes, sensory neurons, and sebaceous glands. While these hair-bearing skin organoids have an inside-out orientation (epidermis facing inwards, dermis exposed outwards) that is the opposite of in vivo skin (epidermis exposed outwards, dermis facing inwards), they formed planar skin and adopted the right orientation when grafted onto the dorsal back of mice [72]. This suggests that signaling cues dictating proper spatial organization of the skin might still be missing from such free-floating culture systems. The inside-out orientation may also be the reason that limited the lifespan of these hair-bearing skin organoids to at most ~150 days, as epidermal stratification with potential shedding of stratum corneum cells, as well as lengthening of the hair shaft, both occur towards the interior core. Nonetheless, taking into consideration the fact that it takes about ~100 days for hair follicles to fully form, a time window of ~50 days to study homeostatic and disease processes involving hair follicles in full anagen may be sufficient. Further, the current technology generates hair-bearing skin organoids with characteristics of face skin and scalp, with the potential to be modified for generating hair-bearing skin organoids that resemble other body sites. 

Despite the limitations of available scaffold-free models, studies using these models have provided much insight into the biology underlying the initial stages of human hair follicle development. As these cultures are relatively easy to set up, the 3D keratinocyte-DP co-cultures and skin organoids are attractive models for high throughput assays for cellular components and bioactives that support hair follicular specification and maturation.

### 3.2. Skin-Mimetic Models

#### 3.2.1. Epidermal-Dermal Skin Construct with DP or Keratinocyte-DP Spheroids

Apart from scaffold-free hair follicle models, DP and/or keratinocyte-DP aggregates have been embedded in 3D skin organotypics in vitro (rather than grafted onto mice in vivo) to provide structural and spatial relevance to enhance the maturation of hair follicular lineages. The basic 3D skin organotypic models are generated with a base layer of dermal fibroblasts embedded in an extracellular matrix (typically collagen), and an overlying layer of keratinocytes which are induced to differentiate into a stratified epidermis at the air–liquid interface [86]. These organotypics, often referred to as “human skin equivalents”, provide a more accurate spatial arrangement of the skin cells, potentially allowing for physiologically relevant directionality in intercellular communications. Indeed, Vahav et al. showed that DP spheroids embedded in the bottom of fibroblast-infused hydrogel expressed chemokines such as CXCL1 and CXCL8, which attracted the downward migration of epidermal keratinocytes in the upper layers of the 3D skin organotypic culture [73]. Notably, the invaginating keratinocytes formed hair follicle-like structures with the engulfed DP spheroids at the base. Similarly, Tan et al. incorporated keratinocyte-DP heterotypic spheroids into 3D organotypics by sequential layering of fibroblasts-, spheroids-, and keratinocytes-infused cell matrixes [74]. The keratinocyte-DP spheroids sandwiched between the dermal and epidermal layers were found to re-organize into downward outgrowths in which CD133+ DP cells were segregated to the base while KRT17+KRT75+ follicle-like cells clustered above them. Cell viability in these outgrowths was also extended beyond the 20 days observed in free-floating spheroids [74]. While no mature hair follicles were generated in these organotypics, these studies demonstrated that spatial cues achieved in these 3D models could drive physiologically relevant cellular reorganization and structural remodeling of the tissues in vitro. 

#### 3.2.2. Epidermal-Dermal Skin Construct with Pre-Patterned Grooves

Building upon the concept of having the right cells in the right place, Abaci et al. made hair follicle-shaped “microwells” in the dermal fibroblast layer of 3D skin organotypics [75]. DP cells were seeded at the base of these microwells and formed aggregates. Keratinocytes were then seeded over the DP aggregates. These keratinocytes grew and differentiated into a hair follicle-like column that engulfed the DP, filled up the microwells, and expressed various hair follicle lineage markers. Strikingly, these hair follicle-like units grew deeper into the dermis and shifted from being perpendicular to a more obtuse angle reminiscent of in vivo skin. A small number of these follicles even produced hair fibers that protruded from the 3D skin organotypic. Of note, foreskin-derived keratinocytes were used to generate these constructs. It is likely that the use of keratinocytes isolated from hair follicles, rather than the foreskin, could improve the rate of hair fiber production in this model.

## 4. Improving Hair Follicle Models

At present, none of the in vitro models fully recapitulate the cellular diversity, intricate structural organization, and compound crosstalk of hair-bearing skin. However, they provide a starting point for enhancements to create better and specific models for investigating hair follicle biology in normal and diseased conditions.

### 4.1. Immune Cells

Immune cells have been conspicuously absent from afore described hair follicle models, be it epidermal-dermal co-culture systems or hair-bearing skin organoids that already have increased cellular complexity [72]. Indeed, immune cells regulate hair follicle function [47,48] and drive inflammatory hair loss, notably AA [21,22]. There have been efforts to incorporate immune cells into 3D skin organotypics, either by embedding immune cells directly into the dermal layer and the insert membrane [87,88] or by perfusing organotypics with immune cells via channels using skin-on-a-chip technologies [89]. In both cases, the immune cells, be they monocyte-derived dendritic cells, CD4+ T cells, or neutrophils, behaved dynamically, migrated through the skin organotypics, and interacted with epidermal keratinocytes. Incorporating disease-relevant immune cell types into skin-mimetic models with hair follicle-like units, or simply co-culturing scaffold-free keratinocyte-DP spheroids and hair-bearing skin organoids with immune cells of interest, could potentially induce further maturation of these various in vitro models.

### 4.2. Vasculature

Current hair follicle models are also void of vasculature. Like immune cells, blood and lymphatic vessels also regulate hair follicle stem cell activity during hair cycling [51,52]. In humans, this regulatory role is suggested in the use of a vasodilator, minoxidil, to treat AGA, although its direct mechanism of action remains to be clarified [90,91,92]. It was demonstrated that dynamic perfusion of nutrients could prolong the lifespan of skin-mimetic constructs and ex vivo hair models cultured in a bioreactor system and skin-on-chip by maintaining optimal levels of nutrients for the tissues [93,94,95]. Nutrient perfusion could be a way to extend the limited lifespan of current in vitro hair follicle models long enough for physiological hair cycling to occur, as well as to fuel the energetically expensive anagen [96]. The flow of the medium through the perfusion system would also facilitate the diffusion of signaling ligands, creating the gradients and effective concentrations of molecules in the intercellular space within the models to mimic the natural tissue microenvironments [93,97,98]. Given that signaling pathways, including WNT, BMP, FGF, and SHH, arise and wean off at different stages of hair follicle development and homeostasis, a built-in vasculature-like system within skin mimetics could allow for precise spatiotemporal control of these various signaling pathways through the perfusion of specific growth factors and inhibitors.

### 4.3. Biomechanical Forces 

Finally, mechanical forces that have been found to govern cellular processes, mediate organ development and homeostasis, and drive pathophysiological processes [99,100,101] could be the key to unlocking the maturation of existing hair peg-like models into hair follicles. In mice, the creation of large full-thickness wounds induces neogenesis of hair follicles that is dependent on the WNT, FGF9, and SHH signaling pathways [12,102,103]. Given that such wound-induced hair follicle neogenesis (WIHN) occurs in the middle, rather than at the periphery, of wounds, and can be triggered only by sufficiently large wounds, but not smaller ones, it is likely that mechanical forces are at play [104]. Indeed, WIHN was found to require a relatively soft microenvironment (characteristic of the center of large wound beds) and was inhibited by increased stiffness (towards the wound edge) [104,105]. This tissue stiffness-dependent phenomenon of WIHN in mice could also explain why DP cells induce de novo hair follicle formation in 3D skin organotypics [106], and why “inside-out” hair-bearing skin organoids flatten into a planar orientation [72], only when grafted onto the dorsal back of mice. It is likely that the in vivo presence of (i) a dermis that is rich in fibroblast-derived ECM and replete with immune cells and vasculature, (ii) a basement membrane (which is typically absent from skin-mimetic models) that tightly adheres the dermis to the overlying epidermis, and (iii) the interfollicular epidermis that bridges individual hair follicles together via their infundibulum collectively create an interconnected network of adhesive forces, either tensile or compressive, to drive various phases of hair follicle development and cycling. In vitro, such topological distribution of mechanical cues could potentially be induced by culturing hair follicle models on stretchable membranes [107,108] and allowing them to work synergistically with molecular signaling pathways manipulated through organ-on-chip technologies to recapitulate the physiological behaviors of hair follicles observed in vivo.

## 5. Conclusions

In summary, despite apparent structural and biological differences between human and mouse skin, we are still heavily reliant on mice to study molecular mechanisms underlying hair and skin diseases, wound healing, and aging. This is due to a lack of comprehensive in vitro human hair-bearing skin models that can largely recapitulate the spatial arrangement, cellular complexity, signaling modules, cell–cell interactions, and mechanical forces of human skin in vivo. Our understanding of human skin biology in development, homeostasis, and disease is now increasing exponentially through global collaborative cell atlasing efforts that employ rapidly advancing single-cell and spatial transcriptomic technologies. It is important to begin integrating this cutting-edge information to innovate more sophisticated human in vitro hair follicle models for translating key critical findings from mice to humans and in the development of therapeutic strategies for hair follicle and skin regeneration.

## Data Availability

Not applicable.

## References

[1] Martel J.L., Miao J.H., Badri T. (2022). Anatomy, Hair Follicle—StatPearls.

[2] Schneider M.R., Schmidt-Ullrich R., Paus R. (2009). The hair follicle as a dynamic miniorgan. Curr. Biol..

[3] Paus R., Cotsarelis G. (1999). The biology of hair follicles. N. Engl. J. Med..

[4] Nishimura E.K., Jordan S.A., Oshima H., Yoshida H., Osawa M., Moriyama M., Jackson I.J., Barrandon Y., Miyachi Y., Nishikawa S.-I. (2002). Dominant role of the niche in melanocyte stem-cell fate determination. Nature.

[5] Tobin D.J. (2011). The cell biology of human hair follicle pigmentation. Pigment. Cell Melanoma Res..

[6] Chang C.-Y., Pasolli H.A., Giannopoulou E.G., Guasch G., Gronostajski R.M., Elemento O., Fuchs E. (2013). NFIB is a governor of epithelial-melanocyte stem cell behaviour in a shared niche. Nature.

[7] Taylor G., Lehrer M.S., Jensen P.J., Sun T.T., Lavker R.M. (2000). Involvement of follicular stem cells in forming not only the follicle but also the epidermis. Cell.

[8] Ito M., Liu Y., Yang Z., Nguyen J., Liang F., Morris R.J., Cotsarelis G. (2005). Stem cells in the hair follicle bulge contribute to wound repair but not to homeostasis of the epidermis. Nat. Med..

[9] Lisse T.S., Sharma M., Vishlaghi N., Pullagura S.R., Braun R.E. (2020). GDNF promotes hair formation and cutaneous wound healing by targeting bulge stem cells. NPJ Regen. Med..

[10] Gonzales K.A.U., Polak L., Matos I., Tierney M.T., Gola A., Wong E., Infarinato N.R., Nikolova M., Luo S., Liu S. (2021). Stem cells expand potency and alter tissue fitness by accumulating diverse epigenetic memories. Science.

[11] Leirós G.J., Kusinsky A.G., Drago H., Bossi S., Sturla F., Castellanos M.L., Stella I.Y., Balañá M.E. (2014). Dermal papilla cells improve the wound healing process and generate hair bud-like structures in grafted skin substitutes using hair follicle stem cells. Stem Cells Transl. Med..

[12] Lim C.H., Sun Q., Ratti K., Lee S.-H., Zheng Y., Takeo M., Lee W., Rabbani P., Plikus M.V., Cain J.E. (2018). Hedgehog stimulates hair follicle neogenesis by creating inductive dermis during murine skin wound healing. Nat. Commun..

[13] Greco V., Chen T., Rendl M., Schober M., Pasolli H.A., Stokes N., dela Cruz-Racelis J., Fuchs E. (2009). A two-step mechanism for stem cell activation during hair regeneration. Cell Stem Cell.

[14] Hsu Y.-C., Pasolli H.A., Fuchs E. (2011). Dynamics between stem cells, niche, and progeny in the hair follicle. Cell.

[15] Higgins C.A., Westgate G.E., Jahoda C.A.B. (2009). From telogen to exogen: Mechanisms underlying formation and subsequent loss of the hair club fiber. J. Investig. Dermatol..

[16] Sawaya M.E., Price V.H. (1997). Different levels of 5alpha-reductase type I and II, aromatase, and androgen receptor in hair follicles of women and men with androgenetic alopecia. J. Investig. Dermatol..

[17] Ellis J.A., Stebbing M., Harrap S.B. (2001). Polymorphism of the androgen receptor gene is associated with male pattern baldness. J. Investig. Dermatol..

[18] Trüeb R.M. (2002). Molecular mechanisms of androgenetic alopecia. Exp. Gerontol..

[19] Hillmer A.M., Hanneken S., Ritzmann S., Becker T., Freudenberg J., Brockschmidt F.F., Flaquer A., Freudenberg-Hua Y., Jamra R.A., Metzenet C. (2005). Genetic variation in the human androgen receptor gene is the major determinant of common early-onset androgenetic alopecia. Am. J. Hum. Genet..

[20] English R.S. (2018). A hypothetical pathogenesis model for androgenic alopecia: Clarifying the dihydrotestosterone paradox and rate-limiting recovery factors. Med. Hypotheses.

[21] Harel S., Higgins C.A., Cerise J.E., Dai Z., Chen J.C., Clynes R., Christiano A.M. (2015). Clinical Medicine: Pharmacologic inhibition of JAK-STAT signaling promotes hair growth. Sci. Adv..

[22] Dai Z., Chen J., Chang Y., Christiano A.M. (2021). Selective inhibition of JAK3 signaling is sufficient to reverse alopecia areata. JCI Insight.

[23] Phillips T.G., Slomiany W.P., Allison R. (2017). Hair Loss: Common Causes and Treatment. Am. Fam. Physician.

[24] Oh J.W., Kloepper J., Langan E.A., Kim Y., Yeo J., Kim M.J., Hsi T.-C., Rose C., Yoon G.S., Lee S.J. (2016). A Guide to Studying Human Hair Follicle Cycling In Vivo. J. Investig. Dermatol..

[25] Van Genderen C., Okamura R.M., Fariñas I., Quo R.G., Parslow T.G., Bruhn L., Grosschedl R. (1994). Development of several organs that require inductive epithelial-mesenchymal interactions is impaired in LEF-1-deficient mice. Genes Dev..

[26] Huelsken J., Vogel R., Erdmann B., Cotsarelis G., Birchmeier W. (2001). beta-Catenin controls hair follicle morphogenesis and stem cell differentiation in the skin. Cell.

[27] Andl T., Reddy S.T., Gaddapara T., Millar S.E. (2002). WNT Signals Are Required for the Initiation of Hair Follicle Development. Dev. Cell.

[28] Chen D., Jarrell A., Guo C., Lang R., Atit R. (2012). Dermal β-catenin activity in response to epidermal Wnt ligands is required for fibroblast proliferation and hair follicle initiation. Development.

[29] Schmidt-Ullrich R., Aebischer T., Hülsken J., Birchmeier W., Klemm U., Scheidereit C. (2001). Requirement of NF-kappaB/Rel for the development of hair follicles and other epidermal appendices. Development.

[30] Zhang Y., Tomann P., Andl T., Gallant N.M., Huelsken J., Jerchow B., Birchmeier W., Paus R., Piccolo S., Mikkola M.L. (2009). Reciprocal requirements for EDA/EDAR/NF-kappaB and Wnt/beta-catenin signaling pathways in hair follicle induction. Dev. Cell.

[31] Biggs L.C., Mäkelä O.J., Myllymäki S.-M., Das Roy R., Närhi K., Pispa J., Mustonen T., Mikkola M.L. (2018). Hair follicle dermal condensation forms via Fgf20 primed cell cycle exit, cell motility, and aggregation. eLife.

[32] Woo W.-M., Zhen H.H., Oro A.E. (2012). Shh maintains dermal papilla identity and hair morphogenesis via a Noggin-Shh regulatory loop. Genes Dev..

[33] Glover J.D., Wells K.L., Matthäus F., Painter K.J., Ho W., Riddell J., Johansson J.A., Ford M.J., Jahoda C.A.B., Klika V. (2017). Hierarchical patterning modes orchestrate hair follicle morphogenesis. PLoS Biol..

[34] St-Jacques B., Dassule H.R., Karavanova I., Botchkarev V.A., Li J., Danielian P.S., McMahon J.A., Lewis P.M., Paus R., McMahon A.P. (1998). Sonic hedgehog signaling is essential for hair development. Curr. Biol..

[35] Chiang C., Swan R.Z., Grachtchouk M., Bolinger M., Litingtung Y., Robertson E.K., Cooper M.K., Gaffield W., Westphal H., Beachy P.A. (1999). Essential role for Sonic hedgehog during hair follicle morphogenesis. Dev. Biol..

[36] Foitzik K., Paus R., Doetschman T., Paolo Dotto G. (1999). The TGF-β2 Isoform Is Both a Required and Sufficient Inducer of Murine Hair Follicle Morphogenesis. Dev. Biol..

[37] Qiu W., Li X., Tang H., Huang A.S., Panteleyev A.A., Owens D.M., Su G.H. (2011). Conditional activin receptor type 1B (Acvr1b) knockout mice reveal hair loss abnormality. J. Investig. Dermatol..

[38] Ouspenskaia T., Matos I., Mertz A.F., Fiore V.F., Fuchs E. (2016). WNT-SHH Antagonism Specifies and Expands Stem Cells prior to Niche Formation. Cell.

[39] Rendl M., Polak L., Fuchs E. (2008). BMP signaling in dermal papilla cells is required for their hair follicle-inductive properties. Genes Dev..

[40] Plikus M.V., Mayer J.A., de la Cruz D., Baker R.E., Maini P.K., Maxson R., Chuong C.-M. (2008). Cyclic dermal BMP signalling regulates stem cell activation during hair regeneration. Nature.

[41] Oshimori N., Fuchs E. (2012). Paracrine TGF-β signaling counterbalances BMP-mediated repression in hair follicle stem cell activation. Cell Stem Cell.

[42] Lien W.-H., Polak L., Lin M., Lay K., Zheng D., Fuchs E. (2014). In vivo transcriptional governance of hair follicle stem cells by canonical Wnt regulators. Nat. Cell Biol..

[43] Hsu Y.-C., Li L., Fuchs E. (2014). Transit-amplifying cells orchestrate stem cell activity and tissue regeneration. Cell.

[44] Kobielak K., Pasolli H.A., Alonso L., Polak L., Fuchs E. (2003). Defining BMP functions in the hair follicle by conditional ablation of BMP receptor IA. J. Cell Biol..

[45] Genander M., Cook P.J., Ramsköld D., Keyes B.E., Mertz A.F., Sandberg R., Fuchs E. (2014). BMP signaling and its pSMAD1/5 target genes differentially regulate hair follicle stem cell lineages. Cell Stem Cell.

[46] Yang H., Adam R.C., Ge Y., Hua Z.L., Fuchs E. (2017). Epithelial-Mesenchymal Micro-niches Govern Stem Cell Lineage Choices. Cell.

[47] Wang E.C.E., Dai Z., Ferrante A.W., Drake C.G., Christiano A.M. (2019). A Subset of TREM2+ Dermal Macrophages Secretes Oncostatin M to Maintain Hair Follicle Stem Cell Quiescence and Inhibit Hair Growth. Cell Stem Cell.

[48] Ali N., Zirak B., Rodriguez R.S., Pauli M.L., Truong H.-A., Lai K., Ahn R., Corbin K., Lowe M.M., Scharschmidt T.C. (2017). Regulatory T Cells in Skin Facilitate Epithelial Stem Cell Differentiation. Cell.

[49] Festa E., Fretz J., Berry R., Schmidt B., Rodeheffer M., Horowitz M., Horsley V. (2011). Adipocyte lineage cells contribute to the skin stem cell niche to drive hair cycling. Cell.

[50] Tanimura S., Tadokoro Y., Inomata K., Binh N.T., Nishie W., Yamazaki S., Nakauchi H., Tanaka Y., McMillan J.R., Sawamura D. (2011). Hair follicle stem cells provide a functional niche for melanocyte stem cells. Cell Stem Cell.

[51] Gur-Cohen S., Yang H., Baksh S.C., Miao Y., Levorse J., Kataru R.P., Liu X., de la Cruz-Racelis J., Mehrara B.J., Fuchs E. (2019). Stem cell-driven lymphatic remodeling coordinates tissue regeneration. Science.

[52] Li K.N., Jain P., He C.H., Eun F.C., Kang S., Tumbar T. (2019). Skin vasculature and hair follicle cross-talking associated with stem cell activation and tissue homeostasis. eLife.

[53] Brownell I., Guevara E., Bai C.B., Loomis C.A., Joyner A.L. (2011). Nerve-derived sonic hedgehog defines a niche for hair follicle stem cells capable of becoming epidermal stem cells. Cell Stem Cell.

[54] Shwartz Y., Gonzalez-Celeiro M., Chen C.-L., Pasolli H.A., Sheu S.-H., Fan S.M.-Y., Shamsi F., Assaad S., Lin E.T.-Y., Zhang B. (2020). Cell Types Promoting Goosebumps Form a Niche to Regulate Hair Follicle Stem Cells. Cell.

[55] Halloy J., Bernard B.A., Loussouarn G., Goldbeter A. (2000). Modeling the dynamics of human hair cycles by a follicular automaton. Proc. Natl. Acad. Sci. USA.

[56] Müller-Röver S., Foitzik K., Paus R., Handjiski B., van der Veen C., Eichmüller S., McKay I.A., Stenn K.S. (2001). A comprehensive guide for the accurate classification of murine hair follicles in distinct hair cycle stages. J. Investig. Dermatol..

[57] Blanpain C., Lowry W.E., Geoghegan A., Polak L., Fuchs E. (2004). Self-renewal, multipotency, and the existence of two cell populations within an epithelial stem cell niche. Cell.

[58] Morris R.J., Liu Y., Marles L., Yang Z., Trempus C., Li S., Lin J.S., Sawicki J.A., Cotsarelis G. (2004). Capturing and profiling adult hair follicle stem cells. Nat. Biotechnol..

[59] Tumbar T., Guasch G., Greco V., Blanpain C., Lowry W.E., Rendl M., Fuchs E. (2004). Defining the Epithelial Stem Cell Niche in Skin. Science.

[60] Ohyama M., Terunuma A., Tock C.L., Radonovich M.F., Pise-Masison C.A., Hopping S.B., Brady J.N., Udey M.C., Vogel J.C. (2006). Characterization and isolation of stem cell-enriched human hair follicle bulge cells. J. Clin. Investig..

[61] Kloepper J.E., Tiede S., Brinckmann J., Reinhardt D.P., Meyer W., Faessler R., Paus R. (2008). Immunophenotyping of the human bulge region: The quest to define useful in situ markers for human epithelial hair follicle stem cells and their niche. Exp. Dermatol..

[62] Inoue K., Aoi N., Sato T., Yamauchi Y., Suga H., Eto H., Kato H., Araki J., Yoshimura K. (2009). Differential expression of stem-cell-associated markers in human hair follicle epithelial cells. Lab. Investig..

[63] Ohnemus U., Uenalan M., Inzunza J., Gustafsson J.-A., Paus R. (2006). The hair follicle as an estrogen target and source. Endocr. Rev..

[64] Langan E.A., Foitzik-Lau K., Goffin V., Ramot Y., Paus R. (2010). Prolactin: An emerging force along the cutaneous-endocrine axis. Trends Endocrinol. Metab..

[65] Sundberg J.P., Beamer W.G., Uno H., Van Neste D., King L.E. (1999). Androgenetic alopecia: In vivo models. Exp. Mol. Pathol..

[66] Crabtree J.S., Kilbourne E.J., Peano B.J., Chippari S., Kenney T., McNally C., Wang W., Harris H.A., Winneker R.C., Nagpal S. (2010). A mouse model of androgenetic alopecia. Endocrinology.

[67] Higgins C.A., Richardson G.D., Ferdinando D., Westgate G.E., Jahoda C.A.B. (2010). Modelling the hair follicle dermal papilla using spheroid cell cultures. Exp. Dermatol..

[68] Higgins C.A., Chen J.C., Cerise J.E., Jahoda C.A.B., Christiano A.M. (2013). Microenvironmental reprogramming by threedimensional culture enables dermal papilla cells to induce de novo human hair-follicle growth. Proc. Natl. Acad. Sci. USA.

[69] Kalabusheva E., Terskikh V., Vorotelyak E. (2017). Hair germ model in vitro via human postnatal keratinocyte-dermal papilla interactions: Impact of hyaluronic acid. Stem Cells Int..

[70] Weber E.L., Woolley T.E., Yeh C.Y., Ou K.L., Maini P.K., Chuong C.M. (2019). Self-organizing hair peg-like structures from dissociated skin progenitor cells: New insights for human hair follicle organoid engineering and Turing patterning in an asymmetric morphogenetic field. Exp. Dermatol..

[71] Lindner G., Horland R., Wagner I., Ataç B., Lauster R. (2011). De novo formation and ultra-structural characterization of a fiber-producing human hair follicle equivalent in vitro. J. Biotechnol..

[72] Lee J., Rabbani C.C., Gao H., Steinhart M.R., Woodruff B.M., Pflum Z.E., Kim A., Heller S., Liu Y., Shipchandler T.Z. (2020). Hair-bearing human skin generated entirely from pluripotent stem cells. Nature.

[73] Vahav I., van den Broek L.J., Thon M., Monsuur H.N., Spiekstra S.W., Atac B., Scheper R.J., Lauster R., Lindner G., Marx U. (2020). Reconstructed human skin shows epidermal invagination towards integrated neopapillae indicating early hair follicle formation in vitro. J. Tissue Eng. Regen. Med..

[74] Tan C.T., Leo Z.Y., Lim C.Y. (2022). Generation and integration of hair follicle-primed spheroids in bioengineered skin constructs. Biomed. Mater..

[75] Abaci H.E., Coffman A., Doucet Y., Chen J., Jacków J., Wang E., Guo Z., Shin J.U., Jahoda C.A., Christiano A.M. (2018). Tissue engineering of human hair follicles using a biomimetic developmental approach. Nat. Commun..

[76] Abreu C.M., Cerqueira M.T., Pirraco R.P., Gasperini L., Reis R.L., Marques A.P. (2021). Rescuing key native traits in cultured dermal papilla cells for human hair regeneration. J. Advert. Res..

[77] Liu Z., Huang J., Kang D., Zhou Y., Du L., Qu Q., Wang J., Wen L., Fu D., Hu Z. (2023). Microenvironmental reprogramming of human dermal papilla cells for hair follicle tissue engineering. Acta Biomater..

[78] Gupta A.C., Chawla S., Hegde A., Singh D., Bandyopadhyay B., Lakshmanan C.C., Kalsi G., Ghosh S. (2018). Establishment of an in vitro organoid model of dermal papilla of human hair follicle. J. Cell Physiol..

[79] Betriu N., Jarrosson-Moral C., Semino C.E. (2020). Culture and Differentiation of Human Hair Follicle Dermal Papilla Cells in a Soft 3D Self-Assembling Peptide Scaffold. Biomolecules.

[80] Quílez C., Valencia L., González-Rico J., Suárez-Cabrera L., Amigo-Morán L., Jorcano J.L., Velasco D. (2024). In vitro induction of hair follicle signatures using human dermal papilla cells encapsulated in fibrin microgels. Cell Prolif..

[81] Jung S.-Y., You H.J., Kim M.-J., Ko G., Lee S., Kang K.-S. (2022). Wnt-activating human skin organoid model of atopic dermatitis induced by Staphylococcus aureus and its protective effects by Cutibacterium acnes. iScience.

[82] Ma J., Liu J., Gao D., Li X., Zhang Q., Lv L., Wang Y., Li J., Zhu Y., Wu Z. (2022). Establishment of Human Pluripotent Stem Cell-Derived Skin Organoids Enabled Pathophysiological Model of SARS-CoV-2 Infection. Adv. Sci..

[83] Ramovs V., Janssen H., Fuentes I., Pitaval A., Rachidi W., Chuva de Sousa Lopes S.M., Freund C., Gidrol X., Mummery C.L., Raymond K. (2022). Characterization of the epidermal-dermal junction in hiPSC-derived skin organoids. Stem Cell Rep..

[84] Li P., Pachis S.T., Xu G., Schraauwen R., Incitti R., de Vries A.C., Bruno M.J., Peppelenbosch M.P., Alam I., Raymond K. (2023). Mpox virus infection and drug treatment modelled in human skin organoids. Nat. Microbiol..

[85] Shafiee A., Sun J., Ahmed I.A., Phua F., Rossi G.R., Lin C.-Y., Souza-Fonseca-Guimaraes F., Wolvetang E.J., Brown J., Khosrotehrani K. (2023). Development of Physiologically Relevant Skin Organoids from Human Induced Pluripotent Stem Cells. Small.

[86] Bell E., Ehrlich H.P., Buttle D.J., Nakatsuji T. (1981). Living tissue formed in vitro and accepted as skin-equivalent tissue of full thickness. Science.

[87] Chau D.Y.S., Johnson C., MacNeil S., Haycock J.W., Ghaemmaghami A.M. (2013). The development of a 3D immunocompetent model of human skin. Biofabrication.

[88] Van den Bogaard E.H., Tjabringa G.S., Joosten I., Vonk-Bergers M., van Rijssen E., Tijssen H.J., Erkens M., Schalkwijk J., Koenen H.J.P.M. (2014). Crosstalk between keratinocytes and T cells in a 3D microenvironment: A model to study inflammatory skin diseases. J. Investig. Dermatol..

[89] Sun S., Jin L., Zheng Y., Zhu J. (2022). Modeling human HSV infection via a vascularized immune-competent skin-on-chip platform. Nat. Commun..

[90] Headington J.T. (1987). Hair follicle biology and topical minoxidil: Possible mechanisms of action. Dermatologica.

[91] Messenger A.G., Rundegren J. (2004). Minoxidil: Mechanisms of action on hair growth. Br. J. Dermatol..

[92] Redmond L.C., Limbu S., Farjo B., Messenger A.G., Higgins C.A. (2023). Male pattern hair loss: Can developmental origins explain the pattern?. Exp. Dermatol..

[93] Ataç B., Wagner I., Horland R., Lauster R., Marx U., Tonevitsky A.G., Azar R.P., Lindner G. (2013). Skin and hair on-a-chip: In vitro skin models versus ex vivo tissue maintenance with dynamic perfusion. Lab Chip.

[94] Kim K., Kim J., Kim H., Sung G.Y. (2021). Effect of α-Lipoic Acid on the Development of Human Skin Equivalents Using a Pumpless Skin-on-a-Chip Model. Int. J. Mol. Sci..

[95] Risueño I., Valencia L., Jorcano J.L., Velasco D. (2021). Skin-on-a-chip models: General overview and future perspectives. APL Bioeng..

[96] Tang Y., Luo B., Deng Z., Wang B., Liu F., Li J., Shi W., Xie H., Hu X., Li J. (2016). Mitochondrial aerobic respiration is activated during hair follicle stem cell differentiation, and its dysfunction retards hair regeneration. PeerJ.

[97] Gomes M.E., Bossano C.M., Johnston C.M., Reis R.L., Mikos A.G. (2006). In vitro localization of bone growth factors in constructs of biodegradable scaffolds seeded with marrow stromal cells and cultured in a flow perfusion bioreactor. Tissue Eng..

[98] Carmona-Moran C.A., Wick T.M. (2015). Transient Growth Factor Stimulation Improves Chondrogenesis in Static Culture and Under Dynamic Conditions in a Novel Shear and Perfusion Bioreactor. Cell. Mol. Bioeng..

[99] Hoffman B.D., Grashoff C., Schwartz M.A. (2011). Dynamic molecular processes mediate cellular mechanotransduction. Nature.

[100] LeGoff L., Lecuit T. (2015). Mechanical Forces and Growth in Animal Tissues. Cold Spring Harb. Perspect. Biol..

[101] Vining K.H., Mooney D.J. (2017). Mechanical forces direct stem cell behaviour in development and regeneration. Nat. Rev. Mol. Cell Biol..

[102] Ito M., Yang Z., Andl T., Cui C., Kim N., Millar S.E., Cotsarelis G. (2007). Wnt-dependent de novo hair follicle regeneration in adult mouse skin after wounding. Nature.

[103] Gay D., Kwon O., Zhang Z., Spata M., Plikus M.V., Holler P.D., Ito M., Yang Z., Treffeisen E., Kim C.D. (2013). Fgf9 from dermal γδ T cells induces hair follicle neogenesis after wounding. Nat. Med..

[104] Harn H.I.-C., Wang S.-P., Lai Y.-C., Van Handel B., Liang Y.-C., Tsai S., Schiessl I.M., Sarkar A., Xi H., Hughes M. (2021). Symmetry breaking of tissue mechanics in wound induced hair follicle regeneration of laboratory and spiny mice. Nat. Commun..

[105] Harn H.I.-C., Chiu P.-Y., Lin C.-H., Chen H.-Y., Lai Y.-C., Yang F.-S., Wu C.-C., Tang M.-J., Chuong C.-M., Hughes M.W. (2022). Topological Distribution of Wound Stiffness Modulates Wound-Induced Hair Follicle Neogenesis. Pharmaceutics.

[106] Thangapazham R.L., Klover P., Wang J.A., Zheng Y., Devine A., Li S., Sperling L., Cotsarelis G., Darling T.N. (2014). Dissociated human dermal papilla cells induce hair follicle neogenesis in grafted dermal-epidermal composites. J. Investig. Dermatol..

[107] Correia Carreira S., Taghavi M., Pavez Loriè E., Rossiter J. (2021). FleXert: A Soft, Actuatable Multiwell Plate Insert for Cell Culture under Stretch. ACS Biomater. Sci. Eng..

[108] Mori N., Morimoto Y., Takeuchi S. (2018). Perfusable and stretchable 3D culture system for skin-equivalent. Biofabrication.

